# The Neurosciences of Health Communication: An fNIRS Analysis of Prefrontal Cortex and Porn Consumption in Young Women for the Development of Prevention Health Programs

**DOI:** 10.3389/fpsyg.2020.02132

**Published:** 2020-08-31

**Authors:** Ubaldo Cuesta, Jose Ignacio Niño, Luz Martinez, Borja Paredes

**Affiliations:** Department of Theories and Analysis of Communication, School of Communications, Complutense University of Madrid, Madrid, Spain

**Keywords:** pornography, neuroscience, health communication, fNRIS, Brodmann area, health prevention campaigns, neuro communications, addiction

## Abstract

This work explores the use of fNIRS neuroimaging technique using young female college students with different levels of consumption of pornography, and the activation of the prefrontal cortex (cue reactivity) when viewing a pornographic clip (cue exposure) versus a control clip. The results indicate that the viewing of the pornographic clip (vs. control clip) causes an activation of Brodmann’s area 45 of the right hemisphere (BA 45, pars triangularis) (*p* < 0.01). An effect also appears between the level of self-reported consumption and the activation of right BA 45: the higher the level of self-reported consumption, the greater the activation (*p* < 0.01). On the other hand, those participants who have never consumed pornographic material do not show activity of the right BA 45 compared to the control clip (*p* < 0.01) indicating a qualitative difference between non-consumers and consumers. These results are consistent with other research made in the field of addictions. It is hypothesized that the mirror neuron system may be involved, through the mechanism of empathy, which could provoke vicarious eroticism. Finally, we suggest the applications that these results may have for primary and secondary prevention programs in the field of problematic consumption of pornography.

## Introduction

In recent years, the technological advances experienced in the field of neuroscience allow us to study the structure of the brain and its functioning from a previously unknown approach. This has been a very important advance in various applied areas of the human sciences. One of the most developed fields thanks to this has been that of public health and prevention since essential research has been generated for the development and optimization of public health interventions (Cuesta-Cambra et al., 2017; Horn et al., 2020).

### Public Health and Prevention

Public and preventive health is an area extraordinary growth. One of the reasons is the cost-benefit ratio it provides. Relatively inexpensive prevention programs manage to reach a large part of the population avoiding risks and diseases that are very expensive to treat once developed. Recent pandemics, especially COVID-19, have further potentiated this area. One of the most relevant areas of public and preventive health is that of addiction, given that it is a problem that affects a large part of the population and with very adverse consequences (Mann et al., 2017).

Currently it is possible to observe neural changes, analyze neuro-images and better understand how the mechanisms that determine the cognitive or behavioral processes of addiction operate. Thanks to these advances, current knowledge about the factors that influence addictive behaviors has taken a qualitative leap, better identifying some of the neurobiological processes through which biological and sociocultural factors contribute to addiction (Volkow and Boyle, 2018). These innovative lines of research play a very relevant role in the design of prevention programs that target more precisely the mechanisms that determine behavioral health problems and, therefore, are essential to prevent behaviors linked to addiction (Fishbein and Dariotis, 2019).

The prevention of addictions consists of persuading large population groups through social persuasion programs developed, fundamentally, through social media. Health communication is a field whose constant evolution confirms that it is a strategic tool that, when used correctly, can be very effective in influencing the behavior of individuals (Goldstein et al., 2015).

The report released by the United States Department of Health and Human Service, Healthy People 2020, highlights the importance of research in the development of health communication programs, an aspect that is confirmed in studies that demonstrate the effectiveness of communication in the prevention of tobacco addictions (Kimber et al., 2020), to gambling (Parham et al., 2019) or to different substances (Timko and Cucciare, 2019).

However, there is currently a lack of basic neurocognitive research that makes it possible to better base prevention and communication programs. Only a deep knowledge of the mechanisms underlying the behavior to be modified will allow the design of adequate preventive programs. In this sense, neuroscience can provide very important data, especially in areas as relevant as addictions, where neurocognitive mechanisms play an essential role.

A communication campaign must be based on the rigor and evidence provided by scientific knowledge (Kumkale et al., 2010), the key being to identify through solid methodologies the neurocognitive factors that intervene in addictions in order to articulate messages that really target those variables that promote a change in behavior. No communication strategy is effective if the messages are not built on a frame of reference that is defined by adequate knowledge of the motivating concepts that provoke the reaction of the brain receptors (Gallagher and Updegraff, 2013). For this reason, some authors speak of the important emergence of a new study area: the neuroscience of persuasion (Cacioppo et al., 2018). As these authors say: “*A growing literature on the neural correlates of persuasion has emerged within the past decade*…*with the majority of studies in this literature focused on the neural correlates of behavior change following exposure to a persuasive appeal*… *but there are important remaining questions to address and major opportunities to be pursued that should attract and ignite research attention*” (Cacioppo et al., 2018, p.165). The importance of integrating the neuroscientific approach with theories of cognitive and emotional information processing underlying persuasive communication and prevention is evident. These investigations will not only increase our knowledge of brain and behavior interaction, but will also allow us to better understand the mechanisms of persuasion and social influence.

### Addiction and Behavioral Addiction

Addiction is one of the biggest public health problems in the contemporary world. The different existing addictions cause a large number of deaths and physical and psychological diseases, also causing disorders of behavior, personality, affectivity and social integration (San Juan, 2019).

In order to comprehend the basic principles of addiction, the most recent studies focus their interest in understanding how the prefrontal lobe works and what are the associated cognitive functions, in order to assess what role dopaminergic reinforcement systems play in the process, inhibitory control, decision making, the search for experiences or social relationships and other factors. Goldstein and Volkow (2002) explain how addiction occurs when the motivational system and the prefrontal inhibitory control system are decompensated and the former gives an exaggerated value to the substance consumed repeatedly while the individual is unable to inhibit a behavior that generates an immediate reward and disregard the risks of this addiction. The increased interest in addictions, more recent research has emerged regarding behavioral addictions. In 2013, the fifth edition of the Diagnostic and Statistical Manual of Mental Disorders was modified to include a subdivision of non-substance-related disorders in the Substance-Related and Addictive disorders category (Goldstein and Volkow, 2002). This subdivision is specifically focused on addictive disorders that do not involve the use of substances and is often called behavioral addictions.

In addition, in recent years the WHO has introduced behavioral addictions into its classification. In this new list, internet addiction is one of the most widespread and could be the cause of important emotional and psychological disorders in the individual (Demetrovics et al., 2008; Vondrácková and Gabrhelík, 2016). Within the internet, the use of the net with the aim of achieving sexual gratification is an increasingly common practice (Cooper and Griffin-Shelley, 2002). There is solid evidence that indicates that problematic consumption of pornography as well as addiction to pornography is increasing, especially in young men (Castro et al., 2019; de Alarcón et al., 2019), causing serious difficulties in this population.

### Pornography Addiction

Thanks to neuroscience, it has been possible to investigate the reasons why adolescents are more likely to develop substance use disorders than adults. The results explain how during adolescence the reward/motivation mechanisms and the limbic-emotional circuits exhibit a state of hyperactivity that fosters greater emotional reactivity and drives the search for behaviors that generate immediate reward. Furthermore, the prefrontal cortex cannot fully self-regulate, leading to increased impulsivity and risk taking (Jordan and Andersen, 2017). Through studies based on neuroimaging methodologies, it has been possible to observe the neural circuits that are activated during addictive behaviors, the gratifying responses, as well as all those processes that activate conditioning to the substance, mood, anxiety or reactivity during withdrawal symptom period (Volkow et al., 2016; Zilverstand et al., 2016).

There are multiple studies based on knowledge of substance-related addictions (Addicott, 2020; Votaw et al., 2020), however, research related to behavioral addiction is much more scarce, highlighting those focused on understanding the relationship between addictive behavior and activation of the Dorsolateral Prefrontal Cortex and its effects on working memory and inhibition of the control of impulsive responses (Alizadehgoradel et al., 2020; Maheux-Caron et al., 2020). One of the behavioral addictions that has attracted the most attention in recent years is pornography addiction. The increased use of the internet may have led to increased consumption and acceptance of pornography (D’Orlando, 2011). Internet pornography is unique as it offers anonymity, free and easy access. These three drivers of internet pornography usage referred to as the “Triple-A engine” are what causes the popularity of internet pornography (Cooper and Griffin-Shelley, 2002). As a consequence of the increased pornography use worldwide, there has been much focus on compulsive internet pornography as a subdomain of hypersexuality (Carroll et al., 2008; Döring, 2009; Griffiths, 2013). On the other hand, individuals who consume cybersex increasingly show younger profiles, and the consumption of online pornography causes a decrease in self-esteem and an increase in stress levels in young people (Ainsworth-Masiello and Evans, 2019). According to the report of the Association for Media Research (AIMC), “Internet Audience March 2020,” 15.3% of users in Spain are young people between 14 and 24 years old, highlighting the progressive growth of included in the section from 14 to 19 years. In addition, the Internet consumption habit by adolescents is characterized by impulsive and uncontrolled behavior in which the need to repeat addictive behaviors prevails, generating a high degree of irritation if browsing is interrupted (Xanidis and Brignell, 2016; Rojas et al., 2018). If we consider that one of the main personality traits of adolescents trained in the digital age is the urgent need to obtain immediate pleasure, we will better understand the activities risk that the consumption of online porn content can pose for individuals still in maturation process of gratification.

Empirical evidence seems to support the notion that abusive online pornography use leads to adverse behavioral (couple-related behavioral changes, reduced social interaction, modified goal standards), physiological (modification of sexual psychophysiological patterns such as erection) and emotional effects (guilt, negative chains of thought, reduction of self-esteem) (de Alarcón et al., 2019). There is also strong evidence that indicates the effects that porn consumption causes on the brain (Muller, 2018). In this way, through reverse induction, it is possible to better understand the mechanisms that underlie the problematic consumption of pornography and even analyze the existence of possible differences or “typologies” profiles of consumers. In this sense, one of the most important differences to investigate in this field is gender differences. Inhóf et al. (2019) have recently presented strong evidence on gender differences in activation of the prefrontal cortex in internet addiction. Sometimes, this behavior ends up becoming a behavioral addiction, which may in turn increase its adverse effects. Behavioral addictions are becoming more prevalent, especially among young adults (e.g., online gambling, excessive smartphone use, and online porn addiction). There exists evidence indicating that women are joining the use of these websites and devices (Shaughnessy et al., 2011, 2017; French and Hamilton, 2018).

On the other hand, health organizations are generating research projects that allow to develop primary and secondary (treatment) prevention programs based on already-existing intervention programs on this field (Vondrácková and Gabrhelík, 2016; Sniewski et al., 2018). Nevertheless, there is no robust empirical evidence on women’s online porn use habits nor on the neurocognitive mechanisms involved in this behavior, which in turn is hurting the creation of these prevention programs.

This research is carried out within the emerging area of “neuroscience of addiction and prevention” (Volkow and Boyle, 2018). In this frame of reference, it has been proposed that the addiction cycle is articulated in three stages and involves three fundamental brain regions: (1) the anticipatory response, mainly caused by stimuli (internal or external) involving the prefrontal cortex and which is responsible of craving, the irrepressible impulse that starts the behavior, (2) the execution of the behavior (with or without substance intake) that involves the base ganglia and the reward circuit, and (3) the extended circuit of the amygdala responsible for withdrawal and restoring balance to the stress response (United States Department of Health and human service, 2016).

The goal of primary prevention is to persuade the target population to prevent the problem behavior from occuring. Therefore, according to this model, the anticipatory response cycle, as responsible for the initiation of the behavior, is the one that plays the most important role. Furthermore, as the model points out, the behavior is activated due to the appearance of a stimulus. Since addictive behavior consists of very powerful learning due to the intensity of the reward, the triggering stimulus acts as a discriminating stimulus. The discriminative stimulus is defined by the psychology of learning as that stimulus that signals to the subject the availability of reinforcement in operant conditioning. When reinforcement involves the dopaminergic brain systems as intensely as it does in addictions, discriminative stimulation and craving play an essential role. This research focuses on studying the importance of the discriminative stimulus consisting of sexually explicit images (cue exposure) and the craving response (cue reactivity) in young women who watch a clip with pornographic content versus a clip with neutral content. This paradigm has been used in the study of substance addictions, such as smoking (Kroczek et al., 2017), but it has not been developed in the field of behavioral addictions such as the consumption of pornography.

Recently, Strahler et al. (2018) have studied the neural correlates of gender differences in distractibility by sexual stimuli. These authors researched neural activity specific to sexual images in brain regions implicated in motivation and reward processing. They found that men as compared to women showed stronger responses in the nucleus caudatus, the anterior cingulate cortex, and the nucleus accumbens. Sexually-motived traits were selectively correlated with nucleus caudatus activity.

The goal of our research is to analyze the role of the dorsolateral prefrontal cortex during pornography cue exposure in young women. By achieving this goal, we intend to provide with knowledge on the neurocognitive fundamentals of this behavior, which will set the foundations of future developments of useful prevention programs. This research may also help to consolidate already-initiated communication programs within health organizations in the field. This study tested whether the prefrontal cortex of participants high (vs. low) in porn consumption showed more activation when exposed to pornographic content relative to a no-treatment condition. In line with previous research (Kühn and Gallinat, 2014; Zangemeister et al., 2019) we expected participants high (vs. low) in porn consumption to increase their activity (cue reactivity) in the prefrontal cortex area when being exposed to footage with pornographic content (cue exposure). We analyzed the activity of the prefrontal cortex using the fNIRS (functional near infrared spectroscopy) technique, which has been shown to be effective in this type of study (Karthikeyan et al., 2020). There is also similar evidence in neuroimaging studies using fNIRS in the field of addictions (Ieong et al., 2019).

As noted, previous research has shown that higher levels of right pars triangularis reactivity as measured by fNIRS in the prefrontal cortex area are associated with self-regulatory endeavors. Of course, it is possible that other stimuli in the lab context might be responsible for such potential difference in activation (e.g., cover story, brain-measuring apparatus, lab environment). Thus, an important goal of the current study was to compare the extent to which the prefrontal cortex activation differs as a function of the type of video (control vs. porn) participants were exposed to. The hypothesis to be investigated proposes that certain areas of the prefrontal cortex will be activated to a greater extent during the viewing of pornography (vs. control). Finally, an interaction effect is also hypothesized: That is, the effect of cue reactivity in the presence of the discriminating stimulus (porn clip) will be greater the higher the rate of pornography consumption and, therefore, the more intense the operant learning has been (Ferrari and Quaresima, 2012). As a research question, the specific prefrontal area where the greatest activation will appear in each circumstance will be considered.

## Materials and Methods

The experimental procedure of the study was approved by the research and ethical protocol commission of the Department of Communication Theories and Analysis of the Complutense University of Madrid.

The research was conducted with 28 subjects: right-handed women, Spanish university students (Mean age = 20.04; SD = 0.79) who voluntarily participated without knowing the objectives of the research. Women of homosexual or bisexual orientation were excluded. In order to control socio-cultural influence, subjects from other countries were also excluded. The activation of the prefrontal cortex was evaluated during the viewing of the clips using the fNIRS system: the 20s pornographic clip was broadcasted followed by a 20s blank screen (porn baseline) and another 20s of a control clip (television interview), followed by a 20s blank screen (baseline control). The order of presentation of the conditions “porn clip + porn baseline” and “control + control baseline” was randomized. The stimuli were designed with the program PsychoPy2^1^, an open source package written in the Python programming language that allows the creation of visual and auditory stimuli, presentation protocols and the registration and analysis of data in a simple way and used for neuroscience and experimental psychology experiments (Peirce, 2007, 2009; Peirce and MacAskill, 2018; Hansen, 2016).

The independent variables were as follows: VI_1_ = video type (porn video vs. control video) and VI_2_ = self-reporting porn consumption as a continuous variable (range 0 to 6). The frontal cortex activation measured with fNIRS was the dependent variable.

### Internet Pornography Use

First, participants were told that they were going to be involved in a study exploring personality variables and reactions to certain stimuli. Participants then responded to some ancillary questions that served to support the cover story, and then responded to the item (e.g., “With what frequency do you usually watch porn per week?”) on which responses ranged from “0” to “6” with higher numbers reflecting more porn consumption indicating if they had viewed pornography. This measure of pornography consumption has been previously used (with a slightly different scale) and has demonstrated its validity and reliability for this type of study (Grubbs et al., 2015).

### Stimulus

During fNIRS recording, subjects were instructed to sit and focus on a blank screen. Then a 20-second clip was then presented, preceded by a 2-second fixation point and followed by a 20-second blank screen as baseline, in an uninterrupted sequence. Once these 20-second of white screen are finished, another 20-second begin with a neutral clip followed by another 20-second of blank screen as a baseline.

To generate a sexual arousal clip, we selected a Roman orgy scene from the film *Caligula*, by Tinto Brass, explicitly depicting sex. For the neutral clip, we chose a standard TV interview with a similar stimulus complexity with the same blank screen as baseline. The choice for a pornographic scene was able to provoke sexual arousal as it was confirmed in a previous pilot study with similar subjects.

In order to avoid cumulative error, the stimuli were presented in random order.

### Measurement of Prefrontal Activity: fNIRS

Data collection using fNIRS was carried out in the neurocommunication laboratory of the School of Communication of the Complutense University of Madrid^2^. Participants were then connected individually to the fNIRS devices to record the prefrontal activity while watching the stimuli.

The prefrontal activity data was recorded using a NIRSport2 fNIRS system by NIRx (NIRx Medical Technologies LLC) which assesses cognitive activation by recording brain oxygenation. Light-emitting diodes (LEDs) in optodes held to the scalp by a tight cap emit light from 650 to 1000 nm. This light passes through the skull and the first layer of the cortex before being picked up by corresponding detectors. Some of this light is absorbed by chromophores, but human tissue is relatively “transparent” in this spectral range (Ferrari and Quaresima, 2012). Hemoglobin, the transport protein that allows red blood cells to carry oxygen, is one such chromophore. A higher concentration of oxygenated hemoglobin results in more light being absorbed. The fNIRS system displays the degree of oxygenation in real-time to researchers based on this principal. The presence of increased oxygenated hemoglobin is interpreted to be the result of more neural resources being used in that area. This is typically referred to as “activation.” Researchers infer cognitive activity based on activation and draw conclusions from there. Other technologies and techniques are also routinely used to assess neural activity. As a neuroimaging technique, fNIRS is a much less expensive alternative to traditional Functional Magnetic Resonance Imaging (fMRI). Despite its lower signal-to-noise (SNR) ratio, fNIRS correlates highly with fMRI measures (Cui et al., 2011), making it a reliable alternative for use in psychophysiological studies. fNIRS is both mobile and less sensitive to movement artifacts than fMRI (Cui et al., 2011), which allows for neuroimaging experiments that would otherwise be impossible, such as full-body motion studies. The ability of fNIRS to be used in a mobile modality is vital to naturalistic studies, since the goal of a naturalistic study is to be as close to real-world activity as possible. For investigations of cue reactivity there are several benefits in using fNIRS, e.g., subjects are sitting in a realistic upright position and can handle real objects to elicit CR by triggering several senses (visual, tactile, olfactory and interoception during movement). Although fNIRS cannot measure hemodynamic activity in subcortical structures, it can assess both the dlPFC involved in inhibitory processes and the OFC involved in the processing of emotional valence (Ehlis et al., 2014).

**FIGURE 1:**
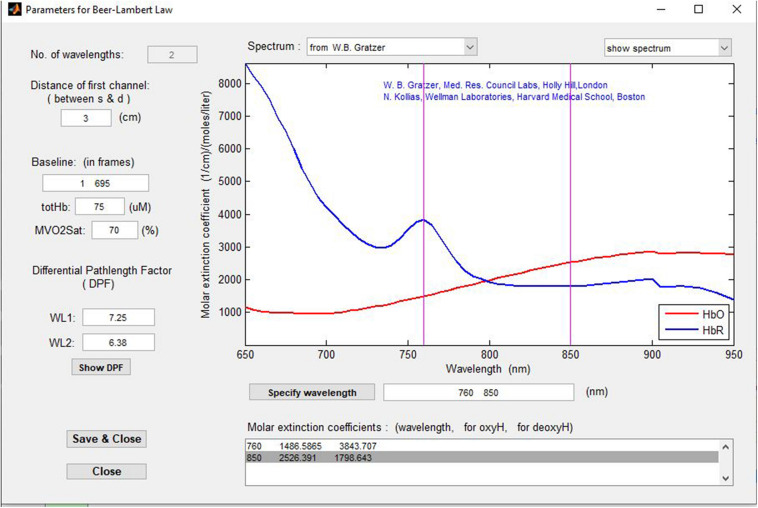
Relative changes in hemoglobin levels.

The fNIRS shows the relative changes in hemoglobin levels, calculated using the modified Beer-Lambert law (Figure 1): oxygenated hemoglobin change: delta O2Hb (μmol/L), deoxygenated hemoglobin change: delta HHb (μmol/L) and total hemoglobin change: delta cHb (μmol/L).

**FIGURE 2:**
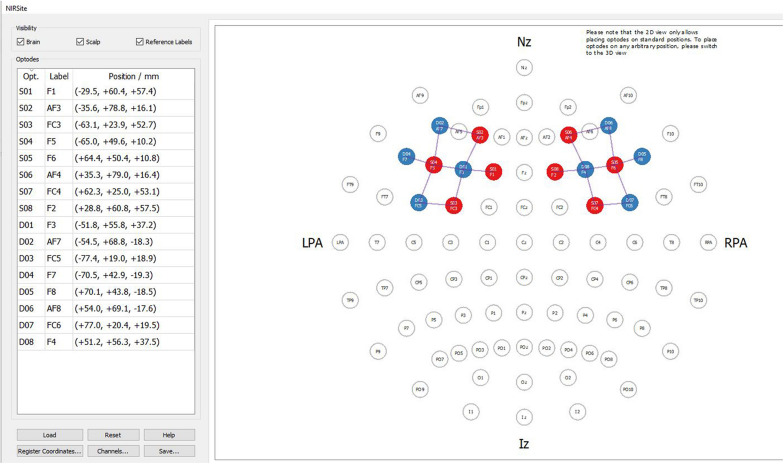
Mounting the optodes to record the signal in the prefrontal cortex.

In order to measure changes in cerebral oxygenation this study utilized the NIRSport2 system. (NIRSport2 8-8, NIRx Medical Technologies LLC, United States) which is a portable, wearable, multichannel fNIRS system consisting of 8 LED illumination sources and 8 active detection sensors. Emitters were placed on positions F1, AF3, FC3, F5, F6, AF4, FC4, and F2 while detectors were placed on positions F3, AF7, FC5, F7, F8, AF8, FC6, and F4 (Figure 2). Eighteen channels were set up covering the prefrontal cortex. The source—detector distance was 3 cm. Optodes were placed on the participant’s head using an Easycap relative to the international 10/20 system (Jasper, 1958). The data was acquired with the Aurora 1.4. acquisition Software (v2014 NIRx Medical Technologies LLC) at two near infra-red light wavelengths of 760 and 850 nm, with a sampling rate of 7.81 Hz.

Participants were then sat in front of a screen, and were told that video footage was going to be shown. They were instructed to watch it while the apparatus measured their brain activity, and to wait for about 20 s after the video was over so a return to baseline could also be collected. After data collection was completed, participants were debriefed, thanked and dismissed.

## Results

The NIRSport2 comes together with Aurora fNIRS: a software platform designed to record the signal. The automated signal optimization algorithm ensures optimal signal quality before a measurement is started. Once data is being recorded, HbO and Hb concentration changes can be visualized in real-time in several display modes. In addition, high-end whole-head visualizations are immediately available.

Also, the nirsLAB package is available: is a MATLAB-based software analysis environment developed to support the study of time-varying near infrared measurements of tissue using the NIRSport2 system. It is composed of modules for: Importing NIRS measurement data. Creating files that contain the optode-position. Preprocessing of measurement data using software programs that exclude data channels having excessive noise, deleting experimentally irrelevant time intervals, removing artifacts from data and filtering to exclude experimentally irrelevant frequency bands. Computing hemodynamic states using wavelength and path length dependent parameter settings. The Data Analysis uses functions found in the SPM (Statistical Parametric Mapping) package to extend the capabilities of nirsLAB to include statistical analysis of hemodynamic-state time series. The functions include: Level-1 general linear model (GLM) analysis of fNIRS hemodynamic-state time series, to evaluate the position-dependent relationships between computed data-channel responses and user-specified temporal models. Level-1 and Level-2 assessment of the statistical significance of the GLM model-fitting coefficients (*t*-test, ANOVA), or of user-defined contrasts of two or more models.

**FIGURE 3:**
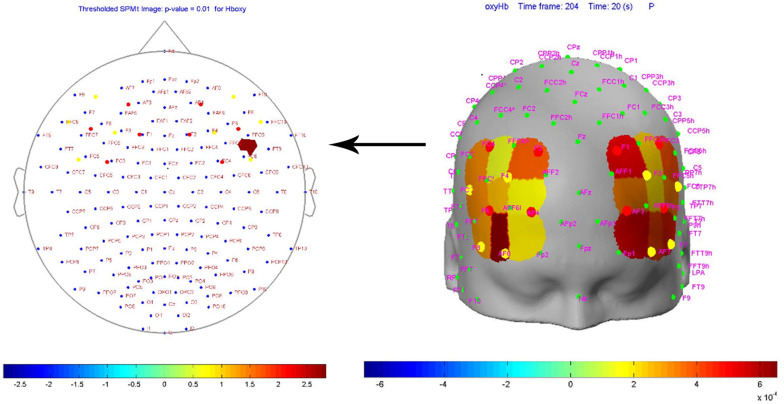
Beta image display at a 0.01 *p*-value of oxygenated hemoglobin (cue reactivity) response to cue exposure (pornography).

**FIGURE 4:**
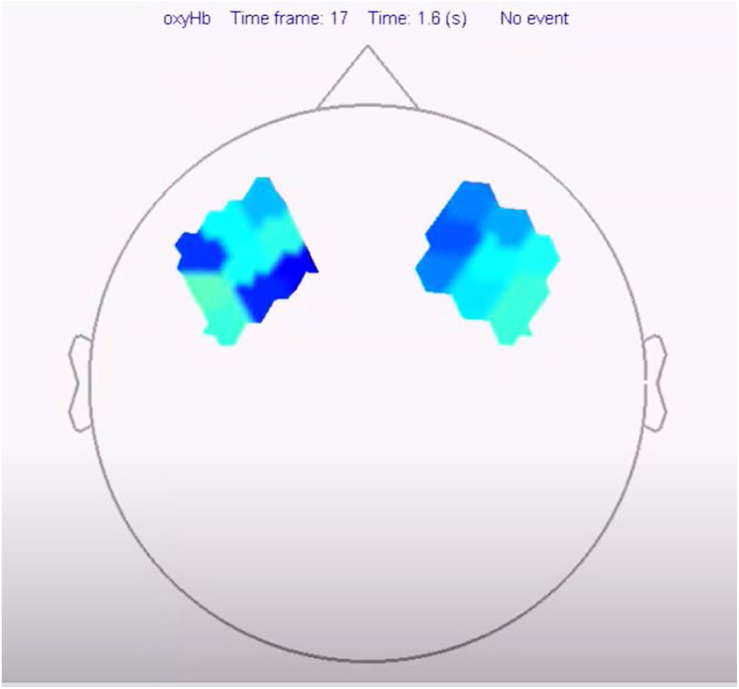
Video activation of prefrontal cortex in a consuming person during the porn clip (Supplementary Video S1).

**FIGURE 5:**
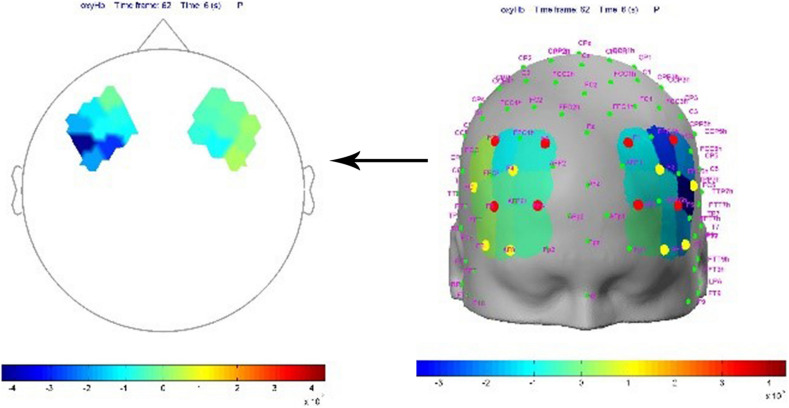
Activation of the prefrontal cortex in a non-consumer when viewing a porn clip.

Figure 3 shows the SPM contrast manager beta image display at a 0.01 *p*-value. The colors denote the magnitude of oxygenated hemoglobin response to pornographic clip vs. non-pornographic clip and the specific area of the prefrontal cortex that was activated (*p*-value = 0.01). The only area that was activated significantly more when viewing the pornographic clip compared to the non-pornographic clip was that corresponding to channel FC6 (optode D07) and F6 (optode S05) corresponding to channel N12 (Figure 3). This channel records right Brodmann area 45 (BA45), more specifically the pars triangularis. In the video that appears as Figure 4, the activation of the prefrontal cortex in a consuming person can be seen dynamically during the viewing of the porn clip. The heatmap in the video shows the highest intensity in the activation of the right BA45^3^. When the subjects were grouped into two clusters (non-consumers vs. consumers) based on the self-report of consumption of pornographic material, the SPM2 analysis produced the same result regarding the activated area (right pars triangularis) verifying the interaction effect (*p* < 0.01): high consumption subjects show more *right* BA45 activity when viewing the pornographic clip than non-consumer subjects (Figure 5). This figure shows how the right activity is lower than the left activity in non-consumers.

**FIGURE 6:**
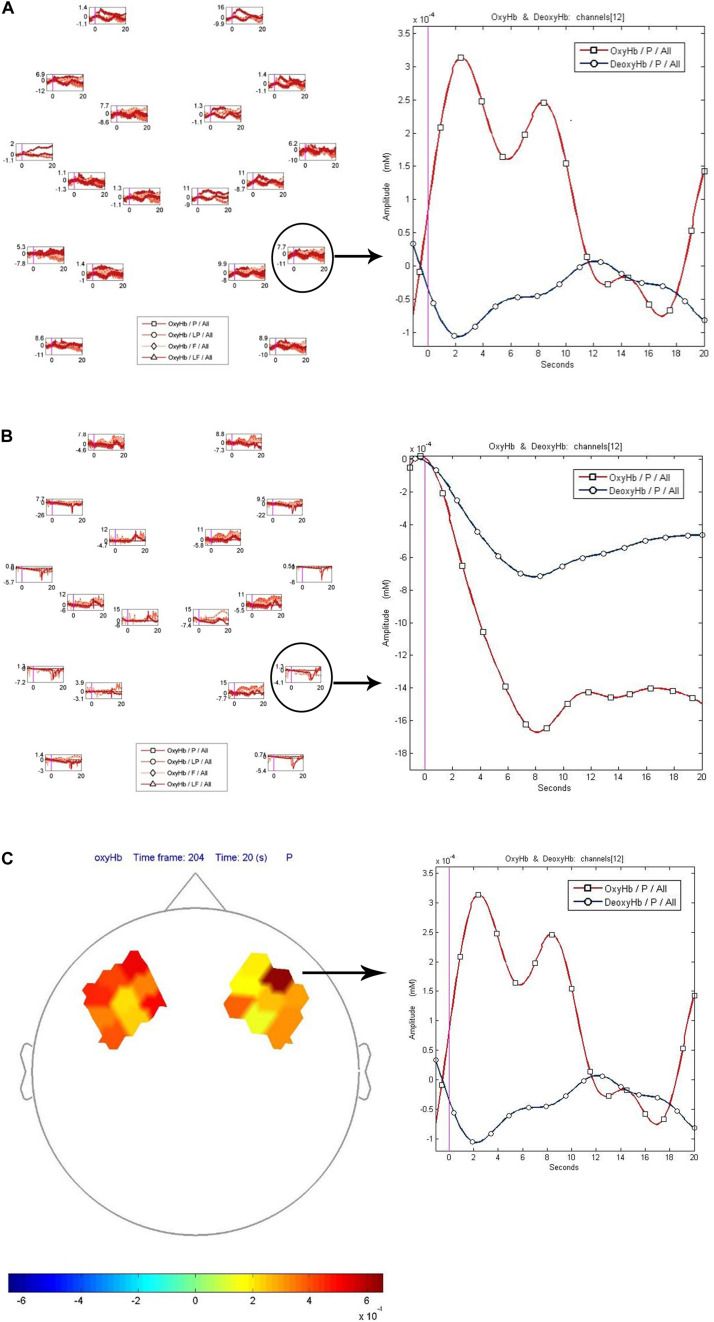
**(A)** Relative changes of hemoglobin in channel 12 in a consumer during the viewing of the porn clip. **(B)** Relative changes of hemoglobin in channel 12 in a non-consumer during the viewing of the porn clip. **(C)** Levels of oxygenated and deoxygenated hemoglobin referred to right BA 45 during porn clip (consumer).

Figures 6A–C show the relative changes in hemoglobin levels *for channel 12* that can be seen during the viewing of the pornographic clip (Figure 6A) in a subject with high consumption scores of pornographic material (consumer) and (Figure 6B) a subject with low consumption scores (non-consumer). In Figure 6C we can see the levels of oxygenated and deoxygenated hemoglobin referred to right BA 45 in consumer during the porn clip.

Once the nirsLAB indicated that the only significant effects appeared in channel 12, we conducted a linear regression analysis using the PROCESS 2.16 macro Model 1 for SPSS (SPSS, RRID:SCR_002865) with porn consumption (centered), porn footage as a multicategorical predictor (Control Video, Return to Baseline Control, Porn Video, Return to Baseline Porn), and the interaction of the two variables on participantś blood flow in the channel 12 of prefrontal cortex (*right pars triangularis*). We contrast coded the porn footage as follows: −2 = Control, −1 = Control Baseline, 1 = Porn Video, 2 = Porn Baseline. In order to properly probe an interaction that has one multi-categorical predictor, we followed the tutorial by Montoya and Hayes (2017). This required transforming the independent variable into three different dichotomous variables (D_1_, D_2_, and D_3_). We report all possible comparisons between conditions (Control vs. Control Baseline, Control vs. Porn, Control vs. Porn Baseline, Control Baseline vs. Porn, Control Baseline vs. Porn Baseline, and Porn vs. Porn Baseline).

**FIGURE 7:**
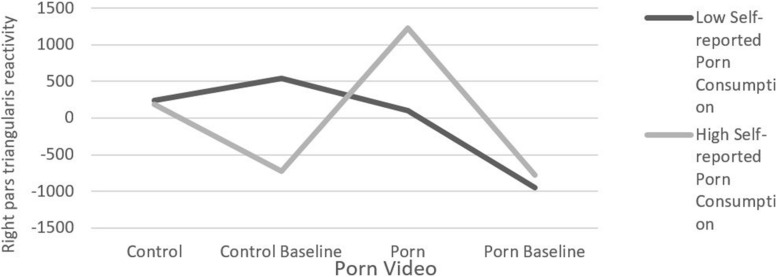
Right pars triangularis reactivity as a function of self-reported porn consumption and porn video.

The regression revealed a significant two-way interaction between porn consumption and video footage, Δ*R*^2^ = 0.019, *F*(3,23427) = 154.67, *p* < 0.001, meaning that the relationship between reported porn consumption and right pars triangularis reactivity varied as a function of the different videos and baselines (see Figure 7 for the entire two-way interaction).

Specifically, when comparing control video and the control baseline, a significant two-way interaction emerged, *B* = −408.79, *t*(23427) = −10.963, *p* < 0.001, 95% CI: −481.881, −335.708. As can be seen in Table 1, there was no relationship between self-reported porn consumption and right pars triangularis reactivity in the control video, *B* = −16.31, *t*(23427) = −0.60, *p* = 0.543, 95% CI: −68.968, 36.337. However, porn consumption was negatively associated with right pars triangularis reactivity in *the control baseline*, *B* = −425.11, *t*(23427) = −16.43, *p* < 0.001, 95% CI: −475.799, −374.422, indicating that participants who reported high (+1 SD) porn consumption showed lower right pars triangularis reactivity than those who reported low (−1 SD) porn consumption.

**TABLE 1:** Right pars triangularis reactivity as a function of self-reported porn consumption and porn video.

		Video			
		Control Video	Control Baseline	Porn Video	Porn Baseline
Self-Reported Porn Consumption	High (+1 SD)	239.95	100.80	539.39	−948.24
	Low (−1 SD)	191.41	1232.25	–725.29	−774.84

A two-way interaction in the opposite direction emerged when comparing *the control video with the porn video*, *B* = 396.634, *t*(23427) = 10.321, *p* < 0.001, 95% CI: 321.309, 471.959. There was no relationship between self-reported porn consumption and right pars triangularis reactivity in the control video, *B* = −16.31, *t*(23427) = −0.60, *p* = 0.543, 95% CI: −68.968, 36.337. However, porn consumption was positively associated with right pars triangularis reactivity in the porn video, *B* = 380.31, *t*(23427) = 13.83, *p* < 0.001, 95% CI: 326.453, 434.184, indicating that participants who reported high (+1 SD) porn consumption showed higher right pars triangularis reactivity than those who reported low (−1 SD) porn consumption.

A similar marginally significant two-way interaction emerged when comparing *the control video with the porn baseline*, *B* = 74.60, *t*(23427) = 1.824, *p* = 0.068, 95% CI: −5.569, 154.772. Specifically, there was no relationship between self-reported porn consumption and right pars triangularis reactivity in the control video, *B* = −16.31, *t*(23427) = −0.60, *p* = 0.543, 95% CI: −68.968, 36.337. However, porn consumption was marginally associated with right pars triangularis reactivity in the porn baseline, *B* = 58.28, *t*(23427) = 1.88, *p* = 0.058, 95% CI: −2.171, 118.743, indicating that participants who reported high (+1 SD) porn consumption showed marginally higher right pars triangularis reactivity than those who reported low (−1 SD) porn consumption.

When comparing *the control baseline with the porn video*, a significant two-way interaction also emerged, *B* = 805.43, *t*(23427) = 21.34, *p* < 0.001, 95% CI: 731.464, 879.394 (Table 2). Reported porn consumption was negatively associated with right pars triangularis reactivity in the control baseline, *B* = −425.11, *t*(23427) = −16.43, *p* < 0.001, 95% CI: −475.799, −374.422. However, porn consumption was positively associated with right pars triangularis reactivity in the porn video, *B* = 380.31, *t*(23427) = 13.83, *p* < 0.001, 95% CI: 326.453, 434.184.

**TABLE 2:** Effects of multiple linear regression with Self-Reported Porn Consumption and Porn Footage (Control Video, Control Baseline, Porn Video, and Porn Baseline) as predictor variables and right pars triangularis reactivity as the dependent variable.

Effects	*B*	*T*
Constant	220.64	4.43**
SRPC	–16.31	–0.60
CV vs. CB	–184.35	−2.69*
CV vs. PV	330.26	4.67**
CV vs. PB	–1099.90	−15.41**
CB vs. PV	514.62	7.49**
CB vs. PB	–915.55	−13.17**
PV vs. PB	–1430.17	−19.99**
SRPC × CV vs. CB	–408.79	−10.96**
SRPC × CV vs. PV	396.63	10.32**
SRPC × CV vs. PB	74.60	1.82
SRPC × CB vs. PV	805.42	21.34**
SRPC × CB vs. PB	483.39	12.00**
SRPC × PV vs. PB	–322.03	−7.79**

*p < 0.05; **p < 0.001. SRPC, Self-Reported Porn Consumption; CV, Control Video; CB, Control Baseline; PV, Porn Video; PB, Porn Baseline.

A significant two-way interaction also emerged between *the porn video and the porn baseline*, *B* = −322.033, *t*(23427) = −7.79, *p* < 0.001, 95% CI: −403.006, −241.060, where porn consumption was positively associated with right pars triangularis reactivity in the porn video, *B* = 380.31, *t*(23427) = 13.83, *p* < 0.001, 95% CI: 326.453, 434.184. However, porn consumption was marginally associated with right pars triangularis reactivity in the porn baseline, *B* = 58.28, *t*(23427) = 1.88, *p* = 0.058, 95% CI: −2.171, 118.743. Lastly, a significant two-way interaction also emerged between the control baseline and the porn baseline, *B* = 483.396, *t*(23427) = 12.00, *p* < 0.001, 95% CI: 404.501, 562.291. As can be seen in Table 1, reported porn consumption was negatively associated with right pars triangularis reactivity in the control baseline, *B* = −425.11, *t*(23427) = −16.43, *p* < 0.001, 95% CI: −475.799, −374.422. However, porn consumption was marginally associated with right pars triangularis reactivity in the porn video, *B* = 58.28, *t*(23427) = 1.88, *p* = 0.058, 95% CI: −2.171, 118.743 (see Figure 7 for the entire two-way interaction).

As can be seen in Table 3, the results of the analysis of variance show statistically significant values at all levels of analysis (*p* < 0.01) for both main effects and interaction, confirming the data previously obtained by the multiple regression.

**TABLE 3:** Effects of two-way ANOVA with Self-Reported Porn Consumption and Porn Footage (Control and Porn) as predictor variables and right pars triangularis reactivity as dependent variable.

Effects	*F*
Intercept	22.73**
SRPC	16.39**
CV vs. PV	35.37**
SRPC × CV vs. PV	33.97**

*p < 0.05; **p < 0.001. SRPC, Self-Reported Porn Consumption; CV, Control Video; PV, Porn Video.

**FIGURE 8:**
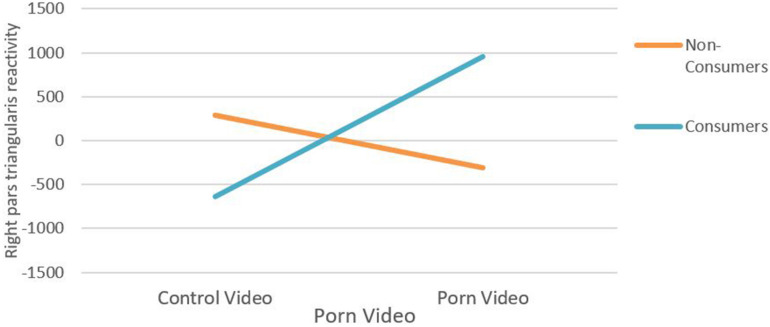
Two-way ANOVA showing right pars triangularis reactivity as a function of extreme values of self-reported Porn Consumption and footage (control vs. porn).

In the following figure (Figure 8) the independent variable “consumption level” has been transformed into a dichotomous variable: subjects who have never consumed pornographic material and subjects who have consumed it. The new dichotomous variable generated two practically identical groups regarding the number of subjects.

The analysis of variance performed (Table 4) indicated that there are main effects (*p* < 0.01) of the factor “type of clip viewed” (control vs. porn) but there are no main effects (*p* < 0.144) of the factor “level consumption” (consumer vs. non-consumer) as well as interaction effect (*p* < 0.01). That is, the interaction effect is strong enough to override the main effect of the type of viewing: subjects who have never seen porn decrease their cortical activation in N12 (BA45, right pars triangularis) while those who have seen it some increase cortical activation significantly in right BA45.

**TABLE 4:** Effects of two-way ANOVA with extreme values of Self-Reported Porn Consumption and Porn Footage (Control and Porn) as predictor variables and right pars triangularis reactivity as the dependent variable.

Effects	*F*
Intercept	1.49
SRPC	2.13
CV vs. PV	18.17**
SRPC × CV vs. PV	87.61**

*p < 0.05; **p < 0.001. SRPC, Self-Reported Porn Consumption; CV, Control Video; PV, Porn Video.

## Discussion

The objective was to find evidence that allows us to contribute knowledge not only to the foundations of neuroscience, but also to the foundations of persuasion neuroscience and communication and health. Thus, the final objective of this research is to find certainties that allow the design of health prevention programs. More specifically, in the area of prevention of problematic consumption of pornographic material by young women, who have recently joined the problematic consumption of pornography (Shaughnessy et al., 2011, 2017; Serrano, 2017; French and Hamilton, 2018).

The increased use of the internet may have led to increased consumption and acceptance of pornography (D’Orlando, 2011). Internet pornography is unique as it offers anonymity, free and easy access. These three drivers of internet pornography usage referred to as the “Triple-A engine” are what causes the popularity of internet pornography (Cooper, 1998). As a consequence of the increased pornography use worldwide, there has been much focus on compulsive internet pornography as a subdomain of hypersexuality (Carroll et al., 2008; Döring, 2009; Griffiths, 2013).

Prevention programs manage to reach a large part of the population avoiding risks and diseases. However, there is an evident lack of neurocognitive research that makes it possible to develop better communication programs in health. Only a knowledge of the mechanisms underlying the behavior to be modified will allow the design of adequate preventive programs.

This research focuses on studying the importance of the discriminative stimulus consisting of sexually explicit images (cue exposure) and the craving response (cue reactivity) in young women consumers and non-consumers of porn videos. This paradigm has been frequently used in the study of substance addictions (Kroczek et al., 2017), but it has been much less developed in the field of behavioral addictions such as the consumption of pornography.

The underlying idea is the following: in order to develop effective prevention programs on porn consumption, it is necessary to know how the discriminative stimuli that trigger the onset of behavior. It is important to consider that the environment in which the behavior of young people develops constantly presents stimuli of high erotic charge that can act as discriminating stimuli. Not only advertising stimuli, but many others, such as those that appear on social networks such as Instagram or TikTok, present a large amount of erotic content that can act as discriminatory stimuli causing behavior and strengthening the learning of problematic behavior. There is also strong evidence that indicates the effects that porn consumption causes on the brain (Muller, 2018). This study tested whether the prefrontal cortex of participants high (vs. low) in porn consumption showed more activation when exposed to pornographic content relative to a no-treatment condition. In line with previous research (Kühn and Gallinat, 2014; Zangemeister et al., 2019) we expected participants high (vs. low) in porn consumption to increase their activity (cue reactivity) in the prefrontal cortex area when being exposed to footage with pornographic content (cue exposure). We analyzed the activity of the prefrontal cortex using the fNIRS (functional near infrared spectroscopy) technique, which has been shown to be effective in this type of study (Ieong et al., 2019; Karthikeyan et al., 2020).

In the current research, 28 young college women self-reported their porn consumption habits and viewed two 20-second video clips (porn vs. control) while the activity of their prefrontal cortex was recorded using fNIRS. The results obtained indicated that the discriminative stimulus caused greater cortical activity in Brodmann’s area 45 (right BA45, pars triangularis) of the right hemisphere in women consumer, but not in non-consuming women (*p* < 0.01). They also indicated that this effect occurs in the experimental group compared to the control group and that the porn stimulus causes a greater effect depending on the degree of consumption. Consistent with our expectations, women who have never consumed pornographic material do not increase the degree of activation of right BA45 compared to the control group. This result is consistent with the interpretation of the porn stimulus as a discriminative stimulus of operant learning “pornography consumption”: if the person has never consumed pornography, the learning has not started, so the stimulus is not discriminatory, but neutral (it could even be aversive). Future research should analyze the difference between “non-consumers” and consumers to test this interpretative hypothesis. In addition, it should be analyzed using different types of addictions such as gambling, social networks, etc. Given that one of the priority interests of this research is to provide evidence for the foundation of prevention programs in health and consumption of pornography in women, it is important to deepen the interpretation of the result: the activation of pars triangularis (area 45 of Brodmann) prefrontal of the right hemisphere. Although this line of research is very recent, there is already some bibliography where more activity of the right pars triangularis has been found in addictions. For example, Irizar et al. (2020) has found that the right lower frontal gyrus volume (i.e., pars triangularis) was significantly larger in both the pathological gambling and cocaine dependency versus control groups. There is an abundant bibliography that links this area with mirror neurons and empathy (Uribe et al., 2019; Krautheim et al., 2019; Rymarczyk et al., 2019). Recently it has been empirically confirmed that the right hemisphere plays an important role in the interpretation of gestures and non-verbal language, especially Brodmann’s area 45 (Inhóf et al., 2019; Krautheim et al., 2019). These data could imply that Brodmann’s area 45, traditionally associated with verbal language in the left hemisphere, is complemented by the functions developed in the right hemisphere. In this way, the left hemisphere would have a role more linked to semantic memory and the understanding of linguistic meanings, while the right hemisphere would deal with the understanding of non-linguistic meanings. Both would work together with working memory but linked to different functions.

On the other hand, neocortical correlates have also been found for the cognitive empathy dimension, while affective empathy would be related to subcortical structures. Functionally, affective empathy appears to be linked to the connectivity between the orbital and cingulate cortices and deeper structures of the limbic system (Uribe et al., 2019; Xiong et al., 2019). A very plausible hypothesis could be that the BA45’s neo-cortical structure acts as an interface between the cognitive and emotional aspects of empathy and the interpretation of the non-verbal behavior of others. Furthermore, this hypothesis is consistent with the fact that a significant number of mirror neurons are found in this area, which would be highly involved in empathy (Gallese, 2001; Decety, 2002; Preston and De Waal, 2002; Decety and Jackson, 2004; Keysers and Gazzola, 2010). In reality, this brain area, and others very close, such as the anterior insula, the anterior cingulate cortex, the inferior frontal cortex, are closely linked to the experience of emotions such as disgust, happiness or pain, especially when viewing another person experiencing these emotions (Botvinick et al., 2005; Lamm et al., 2007). Freedberg and Gallese (2007) have shown the importance of the mirror neuron system for aesthetic experiences. Aesthetic experiences are considered as experiences of perception, creation and evaluation of stimuli that evoke very intense feelings (Chatterjee, 2011; Pearce et al., 2016). Christian Keysers at the Social Brain Lab and colleagues have shown that people who are more empathic according to self-report questionnaires have stronger activations for emotions, providing more direct support for the idea that the mirror system is linked to empathy. It is possible that the mirror system does not passively respond to the observation of actions but is influenced by the mindset of the observer (Molenberghs et al., 2012).

These investigations allow us to propose the following interpretation of the results of our research: the subjects who consume pornography, according to their self-report questionnaires, may show greater empathy for pornographic images. In other words, the “cue exposure” would provoke a greater reaction due to the activation of a kind of “vicarious eroticism” linked to empathy rather than to the pure dopaminergic pleasure provided by brain pleasure systems. Although there is not yet enough empirical evidence, it could be thought that mirror neurons are involved in sexual behavior, especially in its empathic component. White (2019) speaks of “erotic empathy” when referring to this concept. As we have pointed out, this interpretive hypothesis would also be supported by the fact that it is the right cerebral hemisphere that shows BA45 activity. As indicated, the right hemisphere seems to be in charge of processing cognitive interpretations of non-semantic aspects of communication. On the other hand, very clear gender differences have been found in this brain area. For example, Kurth et al. (2017) found significantly larger gray matter volumes in females than males for right BA 44 and BA 45 bilaterally but no significant sex differences with respect to BA 44/45 asymmetry. This could explain the difference between men and women in terms of semantic and empathic capacity in many aspects of psychosocial relationships.

Despite the novelty of this proposal, other authors have found data that supports the idea that right Brodmann’s area 45 of the right hemisphere could be linked to behavioral addictions closely linked to empathy and social relationships. For example, Schmitgen et al. (2020) found that subjects with Smartphone addiction showed greater activation in the right prefrontal cortex, specifically in the pars triangularis (right BA 45). In a very similar sense, Inhóf et al. (2019) has shown that women who self-reported problematic use or addiction to social networks on the Internet showed greater activation in the same area: the pars triangularis (right BA 45) of the right hemisphere and also in the right pars opercularis. Considering that the objective of this work is to contribute knowledge to the area of communication and health neuroscience and, more specifically, of prevention, it is necessary to propose an interpretive hypothesis of these results in terms of the theory of communication and prevention. In this sense, two future research routes can be established. The first is to delve into the difference between “non-consumers” and “consumers”: the data seems to indicate that the reactions to discriminating stimuli (erotic stimuli), responsible for cue exposure, act very differently in non-consumers compared to the rest. In non-consumer participants, right BA 45 (pars triangularis) from the right hemisphere does not appear to be activated, compared to the erotic stimulus, which is very consistent with the idea that it is a discriminative stimulus. The first conclusion, therefore, is important: it is convenient to distinguish very clearly between primary prevention (the subject has not started the problem behavior) and secondary prevention (when the behavior has already started and sought to manage the risks or make it disappear). In the first case, prevention must focus on health education and health promotion programs. Here, the axis of communication should be such that it explains to the subject and their guardians, in the case of minors, the importance of not initiating the behavior. It’s initiation would quickly provoke a sensitization of this cortical prefrontal area, with the consequences of possible craving before discriminative erotic stimuli. In the case of secondary “prevention,” persuasion programs should focus on modifying the attitudes of the subject to eliminate or modify the consumer behavior. In the case of young women, the result of this research seems to indicate that an important motivation in the behavior of consumption of pornography may be the vicarious search for empathic links of an erotic nature that is highly driven by the mirror neuron system. In other words, we would find two variables: the limbic pleasure system characteristic of erotic behavior and the mirror neuron system characteristic of empathic behavior involved.

If these hypotheses are correct, prevention programs in young women should focus on modifying attitudes linked to the search for “erotic empathy” or “vicarious eroticism.” Said in terms of communication theory: the target’s insight indicates that the axis of communication and the strategy of preventive programs should focus on these aspects of human behavior. Therefore (in terms of persuasive social communication theory) the USP (Unique selling proposition) should refer to the benefits in terms of “erotic empathy” that the subject would obtain if they modify their attitudes (and, therefore, their behavior) in this area. In the same sense, the RW (Reason Why) should provide the subject with new reinforcement incentives to replace the cognitive and emotional pleasure provided by “vicarious/empathic eroticism.”

Therefore, in this sense, future lines of research should be developed: analyzing, using neuroimaging techniques (fNIRS, fMRI), how the brain mechanisms of the subjects behave against different preventive communication messages in this area of pornography consumption. The procedure may consist of manipulating, as an independent variable, the type of message, the USP and the RW, using the neuroimaging results as the dependent variable. In this sense, another important line of future research may consist of analyzing gender differences. If the hypothesis is correct, it is reasonable to hypothesize that different areas of the prefrontal cortex are activated in men compared to women, in the face of pornographic stimuli.

The limitations of this research refer to the size of the sample: although the number of subjects is considerable for this type of neuroimaging research, especially considering that the sample is very homogeneous (young Spanish female college students). However, an expansion of the sample size could make it possible to better differentiate between the different degrees of addiction and between “non-consumers” and consumers.

Our paradigm is interesting in several ways. First, it shows that, in young women, BA 45 (pars triangularis) from the right prefrontal cortex plays an important role in the behavior of pornography consumption. This finding could explain the cue reactivity caused by the cue exposure that would be responsible for the craving which, in turn, would trigger consumption behavior. Secondly, these data could be considered a foundation for secondary prevention programs where the communication strategy, the Reason Why and the Unique Selling Proposition were “vicarious/empathic eroticism.” In contrast, for primary prevention programs, the communication strategy should focus on explaining the modifications in the circuits of the right prefrontal cortex that cause the onset of this behavior and its cognitive and emotional consequences. Finally, this research can be useful, if research continues in this direction, to find biological markers in this problematic or addictive behavior, in line with other similar research (Man et al., 2019).

## Data Availability Statement

The raw data supporting the conclusion of this article will be made available by the authors, without undue reservation.

## Ethics Statement

The experimental procedure of the study was reviewed and approved by the commission of investigation and ethical protocol of the Department of Theories and Analysis of Communication of the Complutense University of Madrid. The patients or participants provided their written informed consent to participate in this study.

## Author Contributions

UC aided in the conceptualization and execution of the study, primarily responsible for data analysis and partial drafting of the manuscript and critically reviewed the manuscript and approved its final form. JN aided in the conceptualization of the study, partial drafting of the manuscript, and critically revised the manuscript and approved its final form. LM assisted in data recollection and interpretation, drafting of the manuscript and critical revisions, and approved the final manuscript. BP assisted in data analysis and interpretation and critical review of the manuscript. All authors contributed to the article and approved the submitted version.

## Conflict of Interest

The authors declare that the research was conducted in the absence of any commercial or financial relationships that could be construed as a potential conflict of interest.

## Footnotes

1 www.psychopy.org">http://www.psychopy.org">www.psychopy.org

2 www.neurolabcenter.com

3 https://youtu.be/yB4VNsXICGQ

## References

[B1] Addicott M. A. (2020). “Tobacco addiction: cognition, reinforcement, and mood,” in *Cognition and Addiction. A Researcher’s Guide from Mechanisms Towards Interventions*, Chap. 9, ed. García A. V. (Cambridge, MA: Academic Press), 129–141. 10.1016/B978-0-12-815298-0.00009-5

[B2] Ainsworth-Masiello R. Evans D. T. (2019). Expectations vs reality: in which ways might watching porn online, as male and female adolescents, contribute to poor emotional health? *Educ. Health* 37 109–116.

[B3] Alizadehgoradel J. Nejati V. Sadeghi F. Imani S. Taherifard M. Mosayabi-Samani M. (2020). Repeated stimulation of the dorsolateral-prefrontal cortex improves executive dysfunctions and craving in drug addiction: a randomized, double-blind, parallel-group study. *Brain Stimulation* 13 582–593. 10.1016/j.brs.2019.12.028 32289681

[B4] APA A. (2013). *Guía de Consulta de los Criterios Diagnósticos del DSM-5.*

[B5] Botvinick M. Jha A. P. Bylsma L. M. Fabian S. A. Solomon P. E. Prkachin K. M. (2005). Viewing facial expressions of pain engages cortical areas involved in the direct experience of pain. *Neuroimage* 25 312–319. 10.1016/j.neuroimage.2004.11.043 15734365

[B6] Cacioppo J. T. Cacioppo S. Petty R. E. (2018). The neuroscience of persuasion: a review with an emphasis on issues and opportunities. *Soc. Neurosci.* 13 129–172. 10.1080/17470919.2016.1273851 28005461

[B7] Carroll J. S. Padilla-Walker L. M. Nelson L. J. Olson C. D. McNamara Barry C. Madsen S. D. (2008). Generation XXX: pornography acceptance and use among emerging adults. *J. Adolesc. Res.* 23 6–30. 10.1177/0743558407306348

[B8] Castro J. Gil B. Enrique J. E. Cervigón V. Ballester R. (2019). Signos y síntomas de adicción al cibersexo en adultos mayores. *Int. J. Dev. Educ. Psychol. INFAD Rev. Psicol.* 1 403–412. 10.17060/ijodaep.2019.n1.v4.1596

[B9] Chatterjee A. (2011). Neuroaesthetics: a coming of age story. *J. Cogn. Neurosci.* 23 53–62. 10.1162/jocn.2010.21457 20175677

[B10] Cooper A. (1998). Sexuality and the internet: surfing into the new millennium. *Cyber Psychol. Behav.* 1 187–193. 10.1089/cpb.1998.1.187

[B11] Cooper A. Griffin-Shelley E. (2002). “Introduction. The internet: the next sexual revolution,” in *Sex and the Internet*, ed. Cooper A. (New York, NY: Brunner-Routledge), 1–15.

[B12] Cuesta-Cambra U. Niño-González J. I. Rodríguez-Terceño J. (2017). The cognitive processing of an educational app with EEG and’eye tracking’. *Comunicar*. *Media Educ. Res. J.* 25 41–50. 10.3916/C52-2017-04

[B13] Cui X. Bray S. Bryant D. M. Glover G. H. Reiss A. L. (2011). A quantitative comparison of NIRS and fMRI across multiple cognitive tasks. *Neuroimage* 54 2808–2821. 10.1016/j.neuroimage.2010.10.069 21047559 PMC3021967

[B14] de Alarcón R. de la Iglesia J. Casado N. Montejo A. L. (2019). Online porn addiction: what we know and what we don’t—a systematic review. *J. Clin. Med.* 8:91. 10.3390/jcm8010091 30650522 PMC6352245

[B15] Decety J. (2002). Naturaliser l’empathie. *L’encéphale* 28 9–20. 11963348

[B16] Decety J. Jackson P. L. (2004). The functional architecture of human empathy. *Behav. Cogn. Neurosci. Rev.* 3 71–100. 10.1177/1534582304267187 15537986

[B17] Demetrovics Z. Szeredi B. Rózsa S. (2008). The three-factor model of internet addiction: the development of the problematic internet use questionnaire. *Behav. Res. Methods* 40 563–574. 10.3758/BRM.40.2.563 18522068

[B18] Döring N. M. (2009). The Internet’s impact on sexuality: a critical review of 15 years of research. *Comput. Hum. Behav.* 25 1089–1101. 10.1016/j.chb.2009.04.003

[B19] D’Orlando F. (2011). The demand for pornography. *J. Happiness Stud.* 12 51–75. 10.1007/s10902-009-9175-0

[B20] Ehlis A. C. Schneider S. Dresler T. Fallgatter A. J. (2014). Application of functional near-infrared spectroscopy in psychiatry. *Neuroimage* 85 478–488. 10.1016/j.neuroimage.2013.03.067 23578578

[B21] Ferrari M. Quaresima V. (2012). A brief review on the history of human functional near-infrared spectroscopy (fNIRS) development and fields of application. *Neuroimage* 63 921–935. 10.1016/j.neuroimage.2012.03.049 22510258

[B22] Fishbein D. H. Dariotis J. K. (2019). Personalizing and optimizing preventive intervention models via a translational neuroscience framework. *Prevent. Sci.* 20 10–20. 10.1007/s11121-017-0851-8 29101644

[B23] Freedberg D. Gallese V. (2007). Motion, emotion and empathy in esthetic experience. *Trends Cogn. Sci.* 11 197–203. 10.1016/j.tics.2007.02.003 17347026

[B24] French I. M. Hamilton L. D. (2018). Male-centric and female-centric pornography consumption: relationship with sex life and attitudes in young adults. *J. Sex Marital Ther.* 44 73–86. 10.1080/0092623X.2017.1321596 28441101

[B25] Gallagher K. M. Updegraff J. A. (2013). Health message framing effects on attitudes, intentions, and behavior: a meta-analytic review. *Ann. Behav. Med.* 46:127. 10.1007/s12160-011-9308-7 21993844

[B26] Gallese V. (2001). The’shared manifold’hypothesis. From mirror neurons to empathy. *J. Consciousness Stud.* 8 33–50.

[B27] Goldstein R. Z. Volkow N. D. (2002). Drug addiction and its underlying neurobiological basis: Neuroimaging evidence for the involvement of the frontal cortex. *Am. J. Psychiatry* 159 1642–1652. 10.1176/appi.ajp.159.10.1642 12359667 PMC1201373

[B28] Goldstein S. MacDonald N. E. Guirguis S. (2015). Health Communication and vaccine hesitancy. *Vaccine* 33 4212–4214. 10.1016/j.vaccine.2015.04.042 25896382

[B29] Griffiths M. D. (2013). Social networking addiction: emerging themes and issues. *J. Addict. Res. Ther.* 4:e118 10.4172/2155-6105.1000e118

[B30] Grubbs J. B. Volk F. Exline J. J. Pargament K. I. (2015). Internet pornography use: perceived addiction, psychological distress, and the validation of a brief measure. *J. Sex Marital Ther.* 41 83–106. 10.1080/0092623X.2013.842192 24341869

[B31] Hansen M. (2016). PyVDT: a PsychoPy-based visual sequence detection task. *J. Open Res. Softw.* 4:e22 10.5334/jors.117

[B32] Healthy People (2020). *U.S. Department of Health and Human Services Website.* Available at http://healthypeople.gov/2020/ (accessed June 17, 2020). 10.3109/15360288.2015.1037530 26095483

[B33] Horn S. R. Fisher P. A. Pfeifer J. H. Allen N. B. Berkman E. T. (2020). Levers and barriers to success in the use of translational neuroscience for the prevention and treatment of mental health and promotion of well-being across the lifespan. *J. Abnorm. Psychol.* 129 38–48. 10.1037/abn0000465 31868386 PMC7131983

[B47] Ieong H. F. H. Gao F. Yuan Z. (2019). Machine learning: assessing neurovascular signals in the prefrontal cortex with non-invasive bimodal electro-optical neuroimaging in opiate addiction. *Sci. Rep.* 9 1–14. 10.1038/s41598-019-54316-6 31797878 PMC6892956

[B34] Inhóf O. Zsidó A. N. Perlaki G. Orsi G. Lábadi B. Kovács N. (2019). Internet addiction associated with right pars opercularis in females. *J. Behav. Addict.* 8 162–168. 10.1556/2006.7.2018.135 30663329 PMC7044598

[B35] Irizar P. Albein-Urios N. Martínez-González J. M. Verdejo-Garcia A. Lorenzetti V. (2020). Unpacking common and distinct neuroanatomical alterations in cocaine dependent versus pathological gambling. *Eur. Neuropsychopharmacol.* 33 81–88. 10.1016/j.euroneuro.2020.01.019 32088112

[B36] Jasper H. H. (1958). The ten-twenty electrode system of the International Federation. *Electroencephalogr. Clin. Neurophysiol.* 10 370–375. 10590970

[B37] Jordan C. J. Andersen S. L. (2017). Sensitive periods of substance abuse: early risk for the transition to dependence. *Dev. Cogn. Neurosci.* 25 29–44. 10.1016/j.dcn.2016.10.004 27840157 PMC5410194

[B38] Karthikeyan P. Moradi S. Ferdinando H. Zhao Z. Myllylä T. (2020). Optics based label-free techniques and applications in brain monitoring. *Appl. Sci.* 10:2196 10.3390/app10062196

[B39] Keysers C. Gazzola V. (2010). Social neuroscience: mirror neurons recorded in humans. *Curr. Biol.* 20 R353–R354. 10.1016/j.cub.2010.03.013 21749952

[B40] Kimber C. Frings D. Cox S. Ian P. Albery I. P. Dawkins L. (2020). Communicating the relative health risks of E-cigarettes: an online experimental study exploring the effects of a comparative health message versus the EU nicotine addiction warnings on smokers’ and non-smokers’ risk perceptions and behavioural intentions. *Addict. Behav.* 1 1–8. 10.1016/j.addbeh.2019.106177 31753541 PMC6891257

[B41] Krautheim J. T. Dannlowski U. Steines M. Neziroðlu G. Acosta H. Sommer J. (2019). Intergroup empathy: enhanced neural resonance for ingroup facial emotion in a shared neural production-perception network. *NeuroImage* 194 182–190. 10.1016/j.neuroimage.2019.03.048 30914383

[B42] Kroczek A. M. Haeussinger F. B. Fallgatter A. J. Batra A. Ehlis A. C. (2017). Prefrontal functional connectivity measured with near-infrared spectroscopy during smoking cue exposure. *Addict. Biol.* 22 513–522. 10.1111/adb.12344 26687485

[B43] Kühn S. Gallinat J. (2014). Brain structure and functional connectivity associated with pornography consumption: the brain on porn. *JAMA Psychiatry* 71 827–834. 10.1001/jamapsychiatry.2014.93 24871202

[B44] Kumkale G. T. Albarracín D. Seignourel P. J. (2010). The effects of source credibility in the presence or absence of prior attitudes: implications for the design of persuasive communication campaigns. *J. Appl. Soc. Psychol.* 40 1325–1356. 10.1111/j.1559-1816.2010.00620.x 21625405 PMC3101500

[B45] Kurth F. Jancke L. Luders E. (2017). Sexual dimorphism of Broca’s region: more gray matter in female brains in Brodmann areas 44 and 45. *J. Neurosci. Res.* 95 626–632. 10.1002/jnr.23898 27870461 PMC5119534

[B46] Lamm C. Batson C. D. Decety J. (2007). The neural substrate of human empathy: effects of perspective-taking and cognitive appraisal. *J. Cogn. Neurosci.* 19 42–58. 10.1162/jocn.2007.19.1.42 17214562

[B48] Maheux-Caron V. Trémolière B. Lepage J.-F. Blanchette I. (2020). Transcranial direct current stimulation of the left dorsolateral prefrontal cortex can reduce the detrimental effect of stress on working memory. *Psychol. Neurosci.* 10.1037/pne0000206

[B49] Man M. Nguyen T. S. Battioui C. Mi G. (2019). “Predictive subgroup/biomarker identification and machine learning methods,” in *Statistical Methods in Biomarker and Early Clinical Development*, eds Fang L. Su C. (Cham: Springer), 1–22. 10.1007/978-3-030-31503-0_1

[B50] Mann K. Kiefer F. Schellekens A. Dom G. (2017). Behavioural addictions : classification and consequences. *Eur. Psychiatry* 44 187–188. 10.1016/J.EURPSY.2017.04.008 28646730

[B51] Molenberghs P. Hayward L. Mattingley J. B. Cunnington R. (2012). Activation patterns during action observation are modulated by context in mirror system areas. *Neuroimage* 59 608–615. 10.1016/j.neuroimage.2011.07.080 21840404

[B52] Montoya A. K. Hayes A. F. (2017). Two-condition within-participant statistical mediation analysis: a path-analytic framework. *Psychol. Methods* 22 6–27. 10.1037/met0000086 27362267

[B53] Muller K. J. (2018). Pornography’s effect on the brain: a review of modifications in the prefrontal cortex. *Intuition BYU Undergraduate J. Psychol.* 13:2.

[B54] Parham B. R. Robertson C. Lever N. Hoover S. Palmer T. Lee P. (2019). Enhancing the relevance and effectiveness of a youth gambling prevention program for urban, minority youth: a pilot study of maryland smart choices. *J. Gambling Stud.* 35 1249–1267. 10.1007/s10899-018-9797-4 30121840

[B55] Pearce M. T. Zaidel D. W. Vartanian O. Skov M. Leder H. Chatterjee A. (2016). Neuroaesthetics: the cognitive neuroscience of aesthetic experience. *Perspect. Psychol. Sci.* 11 265–279. 10.1177/1745691615621274 26993278

[B56] Peirce J. MacAskill M. (2018). *Building Experiments in PsychoPy.* Thousand Oaks, CA: Sage.

[B57] Peirce J. W. (2007). PsychoPy—psychophysics software in Python. *J. Neurosci. Methods* 162 8–13. 10.1016/j.jneumeth.2006.11.017 17254636 PMC2018741

[B58] Peirce J. W. (2009). Generating stimuli for neuroscience using PsychoPy. *Front. Neuroinformatics* 2:10. 10.3389/neuro.11.010.2008 19198666 PMC2636899

[B59] Preston S. D. De Waal F. B. (2002). Empathy: its ultimate and proximate bases. *Behav. Brain Sci.* 25 1–20. 10.1017/S0140525X02000018 12625087

[B60] Rojas C. Ramos J. Pardo E. Henríquez F. (2018). Adicción a internet en adolescentes: una breve revisión. *Drugs Addict. Behav.* 3 267–281. 10.21501/24631779.2876

[B61] Rymarczyk K. Żurawski L. Siuda K. J. Szatkowska I. (2019). Empathy in facial mimicry of fear and disgust: simultaneous EMG-fMRI recordings during observation of static and dynamic facial expressions. *Front. Psychol.* 10:701. 10.3389/fpsyg.2019.00701 30971997 PMC6445885

[B62] San Juan P. (2019). Trastornos por consumo de sustancias. *Med. ProgramaFormación Méd. Continuada Acreditado* 12 4984–4992. 10.1016/j.med.2019.09.003

[B63] Schmitgen M. M. Horvath J. Mundinger C. Wolf N. D. Sambataro F. Hirjak D. (2020). Neural correlates of cue reactivity in individuals with smartphone addiction. *Addict. Behav.* 108:106422. 10.1016/j.addbeh.2020.106422 32403056

[B64] Serrano O. (2017). *Tecno-Adicción al Sexo en la Población juvenil: Propuesta De Un Modelo y Una Escala De Evaluación.* Doctoral Thesis, Universidad Complutense de Madrid, Madrid.

[B65] Shaughnessy K. Byers E. S. Walsh L. (2011). Online sexual activity experience of heterosexual students: gender similarities and differences. *Arch. Sex. Behav.* 40 419–427. 10.1007/s10508-010-9629-9 20467798

[B66] Shaughnessy K. Fudge M. Byers E. S. (2017). An exploration of prevalence, variety, and frequency data to quantify online sexual activity experience. *Can. J. Hum. Sex.* 26 60–75. 10.3138/cjhs.261-A4

[B67] Sniewski L. Farvid P. Carter P. (2018). The assessment and treatment of adult heterosexual men with self-perceived problematic pornography use: a review. *Addict. Behav.* 77 217–224. 10.1016/j.addbeh.2017.10.010 29069616

[B68] Strahler J. Kruse O. Wehrum-Osinsky S. Klucken T. Stark R. (2018). Neural correlates of gender differences in distractibility by sexual stimuli. *Neuroimage* 176 499–509. 10.1016/j.neuroimage.2018.04.072 29729394

[B69] Timko C. Cucciare M. A. (2019). “Behavioral health approaches to preventing and treating substance use disorders,” in *Foundations of Behavioral Health*, eds Levin B. Hanson A. (Cham: Springer), 45–70. 10.1007/978-3-030-18435-3_3

[B70] U. S. Department of Health and Human Service (2016). *Surgeon Generalś Report on Alcohol, Drugs and Health.* Available online at: https://addiction.surgeongeneral.gov (accessed June 17, 2020).

[B71] Uribe C. Puig-Davi A. Abos A. Baggio H. C. Junque C. Segura B. (2019). Neuroanatomical and functional correlates of cognitive and affective empathy in young healthy adults. *Front. Behav. Neurosci.* 13:85. 10.3389/fnbeh.2019.00085 31118891 PMC6504763

[B72] Volkow N. D. Boyle M. (2018). Neuroscience of addiction: relevance to prevention and treatment. *Am. J. Psychiatry* 175 729–740. 10.1176/appi.ajp.2018.17101174 29690790

[B73] Volkow N. D. Koob G. F. McLellan A. T. (2016). Neurobiologic advances from the brain disease model of addiction. *N. Engl. J. Med.* 374 363–371. 10.1056/NEJMra1511480 26816013 PMC6135257

[B74] Vondrácková P. Gabrhelík R. (2016). Prevention of internet addiction: a systematic review. *J. Behav. Addict.* 5 568–579. 10.1556/2006.5.2016.085 27998173 PMC5370363

[B75] Votaw V. Pearson M. Stein E. Witkiewitz K. (2020). The addictions neuroclinical assessment negative emotionality domain among treatment-seekers with alcohol use disorder: construct validity and measurement invariance. *Alcohol. Clin. Exp. Res.* 44 679–688. 10.1111/acer.14283 31957027 PMC7069798

[B76] White C. (2019). “Internet pornography: addiction or sexual dysfunction?,” in *Introduction to Psychosexual Medicine*, eds Brough P. A. Denman M. (Abingdon: Taylor & Francis), 189–204. 10.1201/9781315105567-21

[B77] Xanidis N. Brignell C. M. (2016). The association between the use of social network sites, sleep quality and cognitive function during the day. *Comput. Hum. Behav.* 55 121–126. 10.1016/j.chb.2015.09.004

[B78] Xiong R. C. Fu X. Wu L. Z. Zhang C. H. Wu H. X. Shi Y. (2019). Brain pathways of pain empathy activated by pained facial expressions: a meta-analysis of fMRI using the activation likelihood estimation method. *Neural Regen. Res.* 14:172. 10.4103/1673-5374.243722 30531091 PMC6262989

[B79] Zangemeister L. Grabenhorst F. Schultz W. (2019). Neural activity in human ventromedial prefrontal cortex reflecting the intention to save reward. *Soc. Cogn. Affect. Neurosci.* 14 1255–1261. 10.1093/scan/nsaa013 31993656 PMC7137725

[B80] Zilverstand A. Parvaz M. A. Moeller S. J. Goldstein R. Z. (2016). Cognitive interventions for addiction medicine: understanding the underlying neurobiological mechanisms. *Prog. Brain Res.* 224 285–304. 10.1016/bs.pbr.2015.07.019 26822363 PMC5206794

