# Corrigendum: The Neurosciences of Health Communication: An fNIRS Analysis of Prefrontal Cortex and Porn Consumption in Young Women for the Development of Prevention Health Programs

**DOI:** 10.3389/fpsyg.2020.602857

**Published:** 2020-10-06

**Authors:** Ubaldo Cuesta, Jose Ignacio Niño, Luz Martinez, Borja Paredes

**Affiliations:** Department of Theories and Analysis of Communication, School of Communications, Complutense University of Madrid, Madrid, Spain

**Keywords:** pornography, neuroscience, health communication, fNRIS, Brodmann area, health prevention campaigns, neuro communications, addiction

In the original article, the name of one of the authors was incorrectly spelled in the reference for Ieong, H. F. H., Gao, F., and Yuan, Z. (2019). Machine learning: assessing neurovascular signals in the prefrontal cortex with non-invasive bimodal electro-optical neuroimaging in opiate addiction. *Sci. Rep*. 9, 1–14. doi: 10.1038/s41598-019-54316-6 as Leong et al., 2019. It should be Ieong et al., 2019 in all occurrences in the text.

The authors apologize for this error and state that this does not change the scientific conclusions of the article in any way. The original article has been updated.

